# A Randomized Controlled Trial on the Effect of Regular Dart Training on Visual Perception and Attention Level in Pre-Adolescent Children

**DOI:** 10.3390/healthcare12222272

**Published:** 2024-11-14

**Authors:** Çalık Veli Koçak, Umut Canlı, Veli Başal, Monira I. Aldhahi

**Affiliations:** 1 Sports Science Faculty, Aksaray University, Aksaray 68100, Türkiye; velikocak@aksaray.edu.tr (Ç.V.K.); veli.basal@asu.edu.tr (V.B.); 2 Sports Science Faculty, Tekirdag Namik Kemal University, Tekirdag 59030, Türkiye; ucanli@nku.edu.tr; 3 Department of Rehabilitation Sciences, College of Health and Rehabilitation Sciences, Princess Nourah bint Abdulrahman University, P.O. Box 84428, Riyadh 11671, Saudi Arabia

**Keywords:** dart, exercise, adolescent, visual perception, attention

## Abstract

**Background/Objectives:** This study aimed to determine the effects of dart exercises on the visual perception and attention parameters of pre-adolescent students. **Methods**: This study included 40 pre-adolescent secondary school students (n = 20 participants in the exercise group [10 girls and 10 boys] and n = 20 participants in the control group [10 girls and 10 boys]). A pre-test of visual perception and attention was conducted prior to the start of the structured dart exercise program, which lasted 12 weeks. The exercises were performed three days a week, with each session lasting 90 min. After 12 weeks, the visual perception and d2 attention tests were administered to both the exercise and control groups. Repeated measures 2 × 2 analysis of variance (ANOVA) (group × time) was conducted for statistical analysis. **Results**: The results show that there was a statistically significant difference in the group × time interaction for the parameters of visual perception, focusing (E2), concentration (CP), and attention level (TN-E) (*p* < 0.05) Conversely, there was no statistically significant difference in the parameters of psychomotor speed (TN) and selective attention (E1) (*p* > 0.05). In addition to the pre- and post-test scores of the control and exercise groups, it was observed that the exercise group scores showed a significant improvement compared with the control group. **Conclusions:** Based on these findings, it can be concluded that dart exercises can improve the visual perception and attention levels of pre-adolescent secondary school students. These results have implications for the use of dart exercises as a potential cognitive training tool in this age group. Further research could explore the long-term effects and optimal dosage of such a program.

## 1. Introduction

Visual perception, which provides accurate and fast information and is accepted as the first step in information processing, is considered an important factor in children’s verbal and mathematical skills [1,2] and sportive success [3]. Attention, which is defined as the focus of mental activity on a situation [4], is considered an important phenomenon affecting academic performance, while every complicated behavior in sports and situations, such as making quick decisions, deceiving the opponent, focusing on the target, taking position according to the opponent, and reaction speed, requires attention [5]. Although perception and attention are separate processes, they are interrelated. Attention occurs first, but perception interferes with it. Attention is the basic condition for realizing perceptions [6]. However, it has been found that students with visual perception and attention problems have difficulties in motor performance and academic skill acquisition [7].

Participation in structured physical and sports activities is an important method for improving visual perception and attention levels. All activities, including physical education and sports lessons, have been reported to positively affect students’ academic and visual perception skills [8]. It has been determined that children’s perception, attention, and memorization skills improve [9]. In addition, it has been revealed that children who play games that can follow more than one event are successful in games or tasks that require dual attention and improve their motor skills [10,11]. A study conducted to investigate the effect of regular sports practices on the attention of elementary school students found that students who engaged in sports were 83% more careful than those who did not participate in sports [12]. An examination of the literature reveals positive developments in the attention levels of individuals who are active in many branches [13]. In addition, the impact of educational game applications on the attention levels of children aged 9–13 years was investigated. The findings indicated that an eight-week educational game program significantly enhanced attention levels, with students in the exercise group demonstrating higher attention levels than those in the control group [14].

In a study on attentional focus in professional female skiers, attentional ability was investigated in five high-level skiers from the Italian National Women’s Ski Team and ten control participants. Their results show that skiers were more willing to focus faster [15].

Various sports are known to enhance visual perception and attention, including darts being one of them. Darts, in particular, is a valuable sport for developing these skills, as it requires aiming and throwing a small arrow at a circular target [16]. Dart throwing involves sensor, motor, and cognitive components with different movement phases associated with eye–hand coordination, attention, and visual feedback [17]. Power generation and transmission, proprioceptive integration [18], visual perception [19], and attentional focus [20] are critical components in darts. Strategic combinations are used by dart competitors to mentally apply methods [21]. Owing to their ability to reduce spatial mistakes and variability, visual perception and attention are crucial in dart throwing [19,22,23]. Research on darts has shown that visual perception and attention can affect dart throwing accuracy and performance [18,19].

Studies have investigated the effect of dart training on attention in different sampling conditions [24,25,26,27]. These studies indicated that dart training positively affects visual perception and attention. Research has consistently demonstrated the importance of visual perception and attention in dart throwing. Marchant et al. [20] found that novice dart throwers who used controlled attention strategies performed more accurately than those who did not. In different studies, it has been determined that there is a direct relationship between dart throwing accuracy and visual perception and attention. While dart training improves visual perception and attention, the application of visual perception and attention instructions also improves throwing accuracy [25,28].

Although visual perception and attention are important in pre-adolescent children, sufficient research has not yet been conducted, especially in the pre-adolescent population. Research in the literature has mostly been conducted on dart athletes or adult groups. In pre-adolescent children, attention and focus play crucial roles in the learning process. The ability to concentrate and focus enables children to absorb and process information more effectively. Therefore, nurturing and developing attention and focus skills during the pre-adolescent years is vital for healthy cognitive development and lifelong learning.

This study aimed to examine the effects of 12 weeks of regular dart exercises on visual perception and attention in pre-adolescent children using a control group experimental study. This study addresses a significant gap in the existing literature by examining the effects of a specific structured motor activity on key cognitive processes during the critical stage of childhood development. The fact that this intervention will be assessed for its impact on both attention and visual perception underscores the importance of this research, offering valuable insights into the potential of targeted motor exercises to enhance cognitive function in pre-adolescent children.

## 2. Materials and Methods

### 2.1. Research Design and Sampling Procedure

In this study, a parallel two-group pre-test–post-test randomized controlled trial was conducted according to CONSORT guidelines [29]. The study protocol was registered at ClinicalTrials.gov (ID: NCT NCT06574230). Restricted blocked randomization was used, in which each group corresponded, and the participants were randomly assigned to two groups (control and exercise) using a computer. Assignment to groups and randomization were performed without using any concealment mechanism or contact or information with the students. Participants were selected from 212 5th-grade students in 2024.

Ethics committee permission was received for this research from the Aksaray University Human Research Ethics Committee, dated 28 February 2024, with protocol number 2024/01–09. The research procedure was explained to all participants and their parents in detail before the study, and they were given one week to think about it. The parents of all participants signed an informed consent form. Help was received from a 3rd-level dart coach for planning and implementing dart exercises to be used in the project.

### 2.2. Participants

The research group included 45 (girls n = 23, boys n = 22) pre-adolescent secondary school children without any regular experience in dart throwing. However, 40 pre-adolescents (girls, n = 20; boys, n = 20) completed the entire measurement and exercise process. A total of 40 (age = 10.05 ± 1.08) pre-adolescent secondary school students who regularly participated and completed the measurement and exercise process were randomly assigned to the exercise (EG) n = 20 (10 girls, 10 boys) and control (CG) n = 20 (10 girls, 10 boys) groups. The inclusion criteria for this study were as follows: (a) being a 5th-grade student, (b) not having played dart before, (c) not having any history of cardiovascular, neurological, orthopedic, or psychiatric disease, and (d) not taking regular medication.

### 2.3. Experimental Procedure and Data Collecting

Data were collected over two sessions. In the first session, theoretical and practical explanations of the research and testing protocols were provided to the participants. Demographic characteristics, such as age, sex, and medical history, were recorded, and anthropometric measurements, including height and body weight, were obtained. Then, the pre-test data of the visual perception and d2 Test of Attention (d2) were recorded. The tests were administered to the participants in the same order and by the same researchers. The control group did not perform any dart activity during the exercise process. At the end of the 12-week dart training given to the exercise group, the visual perception and d2 attention tests were re-applied to the control and exercise groups, and the final measurement data were obtained.

### 2.4. Visual Perception Test

It is a self-report test developed by Uluç [30] to determine the visual perception levels of children. The test consisted of 13 multiple-choice questions, each with four answer options. The reliability of the test was demonstrated by a Cronbach’s alpha of 0.84, indicating a high reliability. The test duration was approximately 30 min. The test consists of three sub-dimensions: visual discrimination, figure–ground relationship, and figure–ground discrimination. These sub-dimensions contained five, five, and three questions, respectively. The evaluation of the Visual Perception Test was made out of a total of 100 points, with 7.69 points given to each correctly answered question. The lowest score that can be obtained from the test is 0, and the highest score is 100. As the score obtained from the test increases, the visual perception skills of the students also increase.

### 2.5. Attention Test

The attention test is based on the classic d2 Test, which was developed by Brickenkamp [31] and adapted into Turkish by Yaycı [32] and measures selective attention and mental concentration over time. In addition to measuring selective attention, task speed, compliance with rules, and performance quality are sub-features [33]. The test can be applied individually or in groups to individuals between the ages of 9 and 60. The one-page test form contained 14 rows, with 47 in each row, for a total of 658 figures. The letters d and p were used in the test. Some letters have one, two, three, or four dots above or below them. In the test, letters were found in 16 different ways, depending on where they took the dots and their numbers. The main task of the person taking the test was to find the letter d, which had two dots. This can be observed in the tests in three different ways. The test taker had 20 s to perform the task specified in each row. The test completion time was approximately eight minutes. In group applications, an additional 7–8 min is needed outside the task due to giving instructions during the preparation phase, checking that the instructions are understood, and sample application [34]. Two separate scoring keys were used to calculate the test scores. Six points were obtained during testing. These were psychomotor speed (TN; total number of figures marked), selective attention (E1; number of figures skipped without being marked), focusing (E2; number of figures marked incorrectly), concentration (CP; total number of correct lines marked), attention level (TN-E; test performance), and E% (proportion of errors) [32]. The E% parameter was not subjected to research analyses since it did not serve the purposes of the research. In the measurements, the attention level (TN-E) score of the d2 attention test was used as the basis, and the test performance findings were evaluated.

### 2.6. Dart Training Procedure

The training program included a 12-week dart training regimen. The selection of a 12-week period was based on the premise that this duration is adequate for promoting motor skill development and enhancing the attention span in children. This timeframe is commonly employed in similar studies to ensure both the effectiveness of the intervention and the reliability of the collected data [35]. Previous research has demonstrated that meaningful developments can be observed through the implementation of effective training programs, typically spanning a duration of 8–12 weeks. In this regard, shorter periods of time are typically insufficient to achieve statistically meaningful results [36]. In this context, a period of 12 weeks may provide sufficient time for participants to learn and improve their dart skills while also allowing for a more precise and reliable assessment of the effects of visual perception and attention. Therefore, the duration of dart exercise was determined to be 12 weeks in this study.

The exercise group received dart exercises for 12 weeks, 3 days a week, with each exercise lasting 90 min. Within the framework of the 12-week dart training program, the first week included theoretical and practical training on game rules, breathing techniques, and throwing techniques. The second week focused on breathing and throwing techniques. In the third week, the throwing techniques and scoring strategies were examined in detail. In the fourth week, scoring strategies and a specific number of exercises were performed. The fifth week focused on finishing and exiting dart techniques. In the sixth week, a specific number of studies and competition practices were conducted. In the seventh week, a specific number of studies that were completed in darts were covered. In the eighth week, the first set of studies, exit techniques, and competitive practices were conducted. In the ninth week, finishing dart and competition practices were conducted. In the tenth, eleventh, and twelfth weeks, only competitions were organized.

### 2.7. Statistical Analysis

SPSS (SPSS Inc., version 28, Chicago, IL, USA) was used for data analysis, and the significance level was set at *p* < 0.05. Before any analysis, the homogeneity of the data was assessed using Levene’s test, and normality was assessed using the Shapiro–Wilk test. Descriptive statistics are reported as means and standard deviations. A two-factor repeated-measures analysis of variance (2 × 2 ANOVA) was applied (group × measurement). Box’s Test for Equality of Covariance Matrices was used to determine whether there were significant differences in the covariances in the measurement groups between paired combinations of groups. In addition, the magnitude of the differences between the groups was assessed using the partial eta squared (*η_p_*^2^) effect size. The partial eta squared shows the extent to which the variance of the dependent variable is explained by the independent variable [37]. According to Cohen [38], an eta square value between 0.01 and 0.06 can be interpreted as a small effect, an eta square value between 0.06 and 0.14 can be interpreted as a medium effect, and an eta square value of 0.14 and above can be interpreted as a large effect.

## 3. Results

Table 1 presents the characteristics of the participants. The average age was 10 years, the mean height of participants was 141.8 cm (±1.20 cm), and the mean weight was 38.8 kg (±4.04 kg).

The comparison of pre-test and post-test averages of the visual perception and attention of the exercise and control groups is detailed in Table 2.

As shown in Table 2, the post-test means for visual perception, psychomotor speed (TN), focusing (E2), concentration (CP), and attention level (TN-E) were higher in the exercise group. In the control group, an increase was observed in the mean psychomotor speed (TN), focusing (E2), concentration (CP), and attention level (TN-E).

Two-way repeated ANOVA was performed to determine the changes in the participants’ visual perception and attention parameters over time (Table 3).

In terms of the exercise and control groups were considered as a whole group, significant differences were found between the mean post-test scores of visual perception (F _(1–38)_ = 139.799; *p* < 0.001), TN (F _(1–38)_ = 82.890; *p* < 0.001), E1 (F _(1–38)_ = 11.095; *p* < 0.01), CP (F _(1–38)_ = 70.723; *p* < 0.001), and TN-E (F _(1–38)_ = 227.699; *p* < 0.001) and the mean pre-test scores of this large group. There were no significant differences in E2 levels.

In the two-way analysis of variance conducted to determine whether being in the exercise group had a significant effect on test scores for mixed measurements, the group–time interaction showed that the exercise group’s score increase was significantly higher than that of the control group in visual perception (F _(1–38)_ = 40.227; *p* < 0.05), E2 (F _(1–38)_ = 10.669; *p* < 0.05), CP (F _(1–38)_ = 16.456; *p* < 0.05), and TN-E (F _(1–38)_ = 5.608; *p* < 0.05). However, no statistically significant difference was found in the group–time interaction comparison of the psychomotor speed parameters TN and E1.

## 4. Discussion

The findings of this study revealed that regular dart training had a significant positive impact on visual perception and attention parameters, including selective attention (E1), concentration (CP), and attention level (TN-E) within the exercise group. These findings suggest that regular participation in dart exercises enhances visual perception and attention levels among pre-adolescent children.

In the context of our study, we found that regular dart exercise had a positive impact on the visual perception of pre-adolescent children. This finding is supported by that of a previous study. For instance, Haghkhah et al. [24] observed that mental imagery exercises improved attention deficits among 33 male students, indicating a beneficial effect of targeted mental activities on attentional capacities. Abdollahipour et al. [25] further emphasized the importance of visual feedback in dart performance, demonstrating that an external focus during dart throwing improved performance, particularly in transfer tests, across a sample of 100 participants. Similarly, Neugebauer et al. [26] showed that dart training with a focus on visual perception significantly increased quiet eye duration—a key marker of enhanced visual perception—in a week-long training program involving four groups. This result aligns with that of Bahrami et al. [27], who found that using grand visual illusions combined with an external focus of attention improved motor learning among 40 beginner athletes. Additionally, Sherwood et al. [39] reported that, as the number of dart practice trials increased, errors related to visual perception and attention decreased, highlighting the cumulative benefits of repetitive practice on these cognitive functions. These studies complement our findings by underscoring the role of dart exercises and visual feedback in enhancing visual perception and attention, particularly through mechanisms such as focused attention, visual feedback, and repetitive practice.

Our study also found that regular dart exercises positively affected the attention levels (TN-E) of pre-adolescent children, a finding consistent with prior research. Yönal et al. [40] reported similar outcomes, demonstrating that dart training enhanced attention levels in a sample of 30 students aged 9–13 years. Vatansever [41] further showed that middle school students who practiced darts at least three times per week experienced a more substantial reduction in attention deficits than those who did not participate. Additionally, Varol and Türkmen [42] studied 20 students aged 11–13 years with initially low attention levels, noting significant improvements in attention among those in the exercise group. In support of these findings, Norouzi et al. [43] observed increased focus in 30 novice dart athletes following only four training sessions. Collectively, these studies reinforce the positive effects of dart exercise on attention levels in young individuals.

A primary limitation of this study is the focus on pre-adolescent children, which makes it challenging to directly compare with studies conducted on adult populations. However, it is still instructive to examine the existing literature on exercise and visual perception in different age groups. For instance, Salehi et al. [44] demonstrated significant improvements in visual perception among women aged 21–26 after a 6-week exercise program. Similarly, a controlled study of 43 university students found improved attention performance in the intervention group. These findings suggest that regular exercise may enhance visual processing in young adults. In the realm of sports, Law and Wong [45] observed that 61 dart athletes employing an internal focus of attention had lower attention deficits compared with those using an external focus. This suggests that the specific attentional demands of a sport, rather than exercise per se, may influence the direction of the effects on visual attention. Furthermore, Nabizade and Hosseini [46] found that 20 dart training sessions with 40 university students new to dart throwing increased their attention levels. This indicates that the acquisition of sport-specific skills, which require focused visual attention, may also confer benefits to attentional functioning. Although differences in age, frequency, and duration of training exist across these studies, the overall pattern suggests that both regular exercise and sport-specific training can lead to positive visual perception and attention outcomes. Future research should aim to clarify the specific mechanisms underlying these effects and determine whether they generalize across the lifespan.

A unique strength of this study lies in its novel focus on the effects of regular dart exercises on visual perception and attention in pre-adolescent children, an age group that has been understudied in this area. The structured training program, consisting of 90 min per session, three times per week, for 12 weeks, adds robustness to this study’s findings. The results provide preliminary evidence that incorporating dart exercises into daily activities for pre-adolescent children can enhance their visual perception and attention. This program also serves as an effective model for physical therapists and physical education teachers seeking to implement sports training that can benefit their cognitive development. However, this study’s design limits insight into the chronic, long-term effects of dart training, emphasizing the need for extended follow-up to evaluate these outcomes. The limited research on this age group further complicates comparisons with the existing literature, which has primarily focused on adult populations.

This study has several limitations. First, the impact of fatigue on visual perception and attention was not considered despite the physically demanding nature of the dart training program. Fatigue can significantly affect attentional focus and visual processing speed, and future studies should incorporate measures of fatigue to better understand its effects. Second, psychological factors (e.g., stress and motivation) that may influence attention and visual perception were not evaluated. These factors can play a crucial role in mediating the effects of exercise on cognition, and their assessment is recommended for future research. Finally, this study applies to pre-adolescent children, which limits the generalizability of the findings to other subsets of the population.

## 5. Conclusions

The results of the current study suggest that regular participation in dart exercises enhances visual perception and attention levels among pre-adolescent children. The results align with a limited number of existing studies, demonstrating that pre-adolescent children who engage in regular dart training exhibit higher levels of visual perception and attention than their non-participating peers. Nonetheless, regular dart training appears to significantly improve visual perception and attention in pre-adolescents.

Future studies should explore diverse samples and variables to investigate the potential effects of fatigue and psychological factors on visual perception and attention, which could substantially enhance the existing literature. Research on children of varying ages can also provide a more comprehensive perspective. Studies examining long-term effects may need to increase exercise frequency and duration. Additionally, qualitative research methods could provide in-depth insights into this field.

Based on these findings, integrating dart exercises into daily routines for pre-adolescent children could enhance visual perception and attention. Providing access to dart sports in schools could amplify these benefits across a wider child population.

## Figures and Tables

**Table 1:** Characteristics of participants.

Variable	Mean ± SD
Age (y)	10 ± 1
Height (cm)	141.8 ± 1.20
Weight (kg)	38.8 ± 4.04
Sex, n (%)	
Girls	20 (50%)
Boys	20 (50%)

**Table 2:** Within-group change in visual perception and attention.

Variables	Pre-Test		Post-Test	
	Exercise Group	Control Group	Exercise Group	Control Group
Visual Perception	14.22 ± 9.09	10.76 ± 6.78	56.90 ± 16.24	8.07 ± 6.34
Psychomotor Speed (TN)	412.15 ± 89.48	410.65 ± 128.56	586.05 ± 76.93	497.0 ± 110.20
Selective Attention (E1)	66.35 ± 55.25	41.55 ± 44.06	41.80 ± 30.78	32.45 ± 34.21
Focusing (E2)	19.85 ± 21.64	42.60 ± 52.39	23.25 ± 21.88	47.75 ± 50.31
Concentration (CP)	155.15 ± 49.64	128.50 ± 50.56	247.15 ± 50.01	166.95 ± 3.53
Attention Level (TN-E)	325.95 ± 72.33	321.50 ± 76.07	531.00 ± 90.74	416.80 ± 73.20

**Table 3:** A model analysis examining the effects of the intervention.

Variables	Group	F	*p*-Value	*η_p_* ^2^	Group × Measurement			
	Mean Square				Mean Square	F	*p*-Value	*η_p_* ^2^
Visual Perception	7995.201	139.799	0.001 ***	0.786	22.49	40.227	0.000 *	0.65
Psychomotor Speed (TN)	338,650.313	82.890	0.001 ***	0.686	476.46	2.695	0.105	0.96
Selective Attention (E1)	5661.613	11.095	0.02 **	0.226	45.54	3.203	0.077	0.68
Focusing (E2)	10.513	0.017	0.898	0.000	30.86	10.669	0.002 *	0.51
Concentration (CP)	85,086.012	70.723	0.001 ***	0.650	174.44	16.456	0.000 *	0.93
Attention Level (TN-E)	451,050.612	227.699	0.001 ***	0.857	398.81	5.608	0.020 *	0.96

* Denotes *p*-value < 0.05. ** Denotes *p*-value < 0.01. *** Denotes *p*-value < 0.001. Bold text indicates statistically significant differences.

## Data Availability

Data will be made available upon request to the corresponding author.
